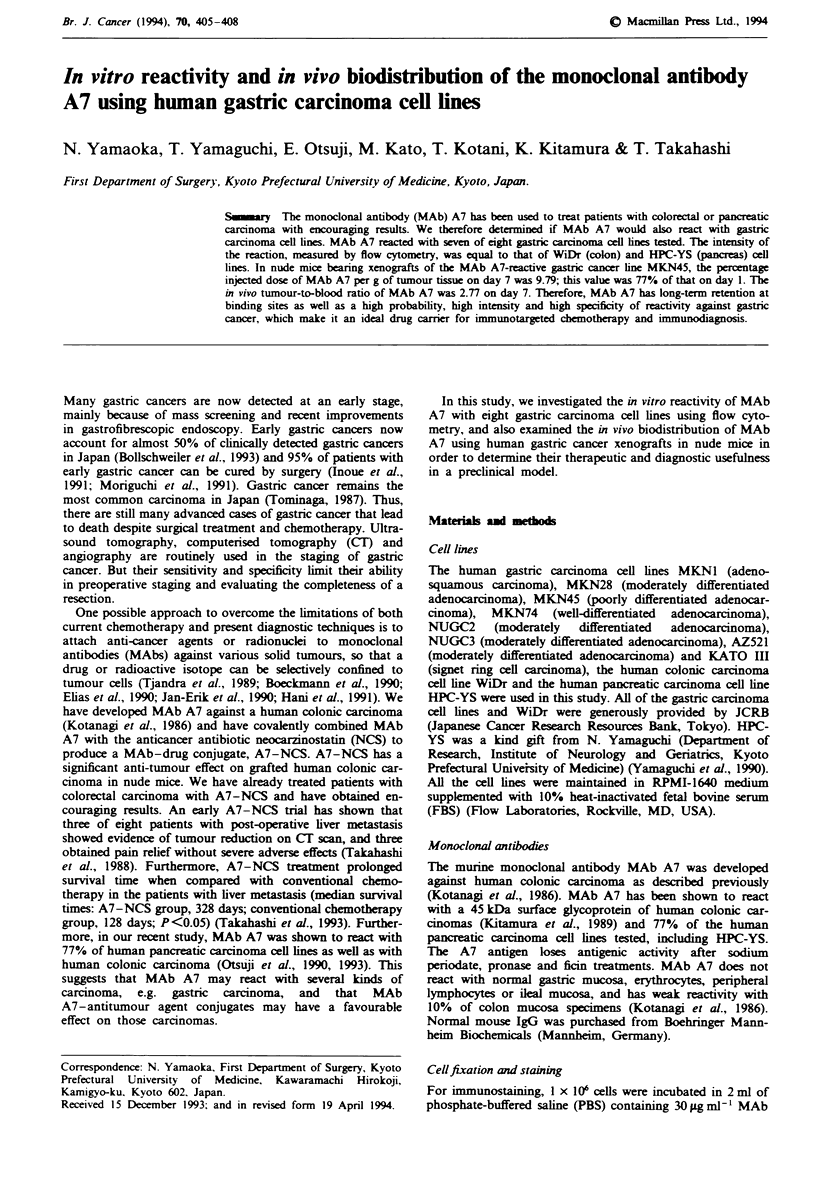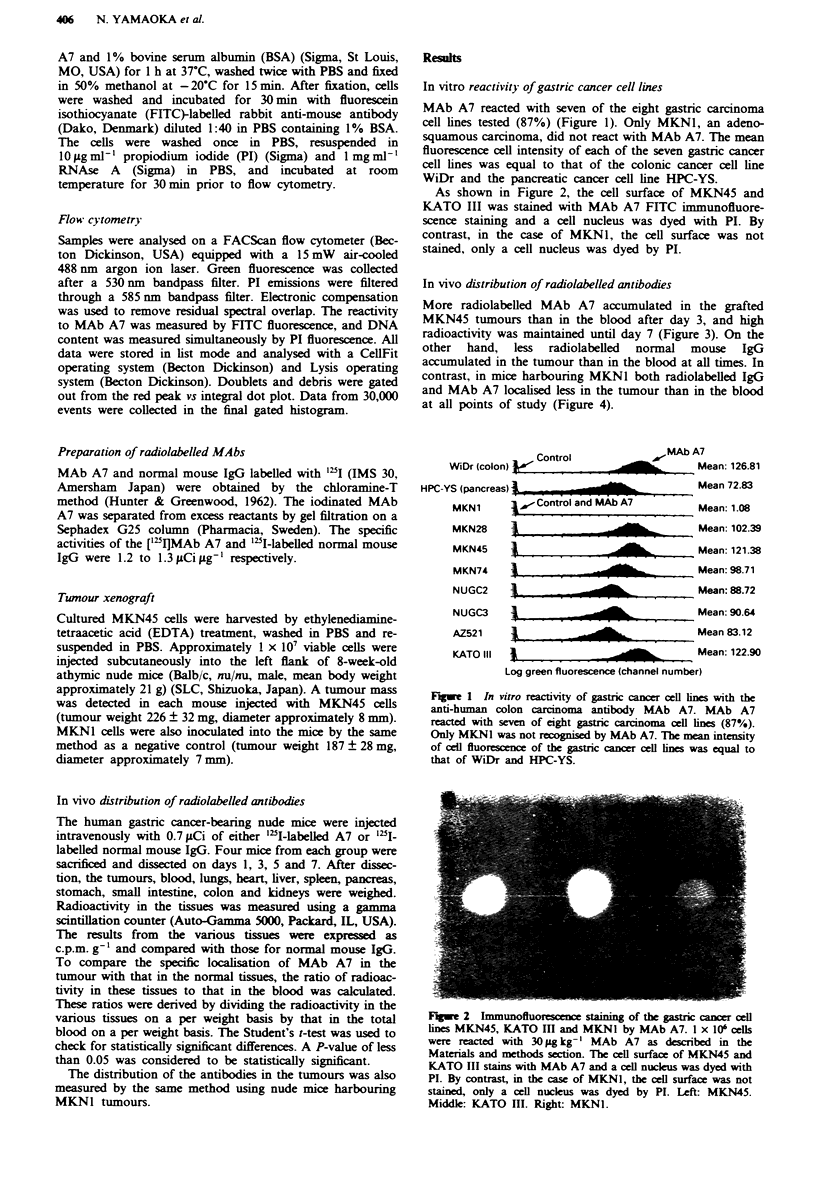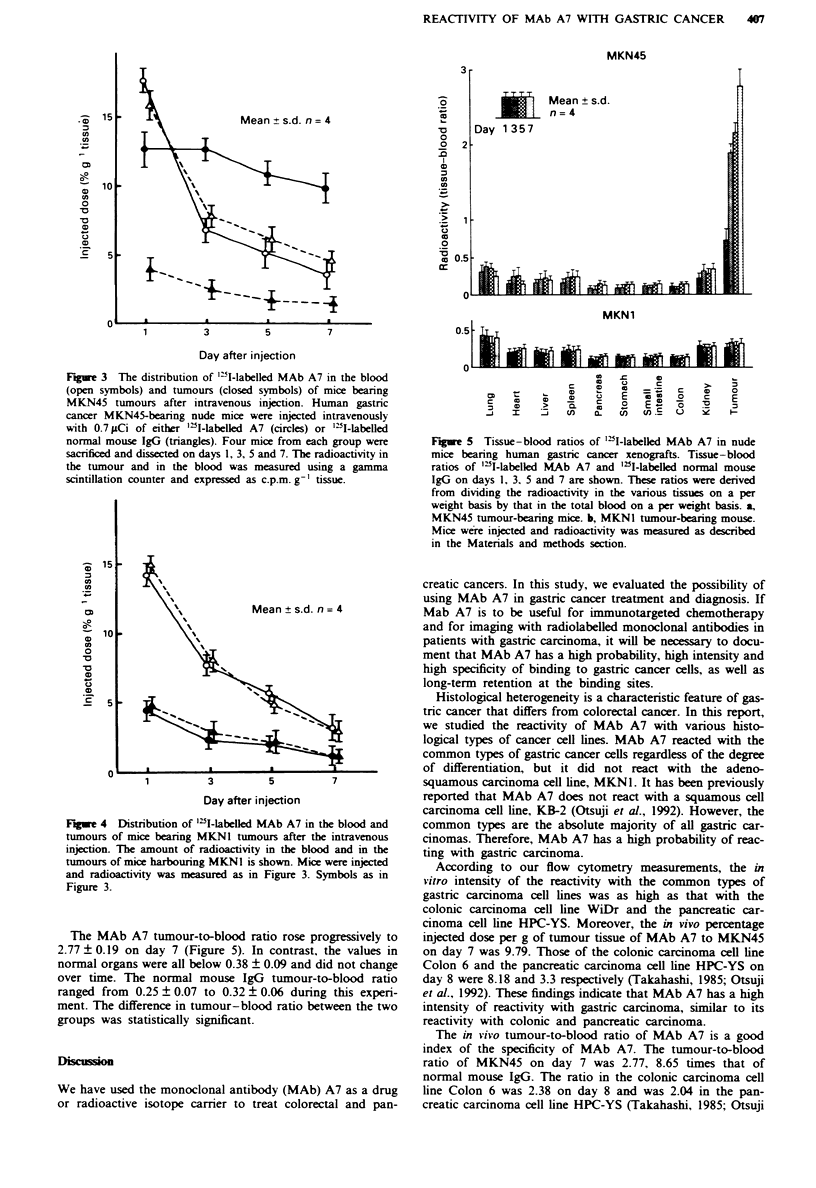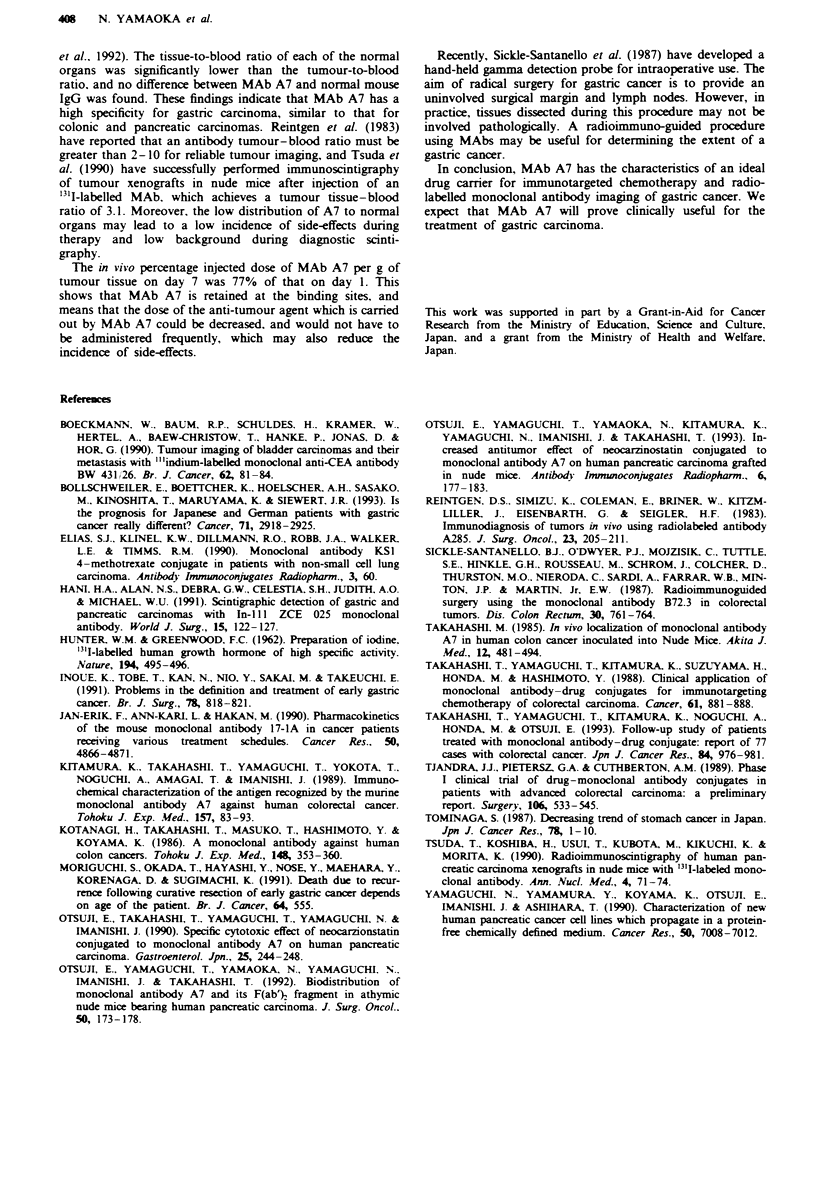# In vitro reactivity and in vivo biodistribution of the monoclonal antibody A7 using human gastric carcinoma cell lines.

**DOI:** 10.1038/bjc.1994.318

**Published:** 1994-09

**Authors:** N. Yamaoka, T. Yamaguchi, E. Otsuji, M. Kato, T. Kotani, K. Kitamura, T. Takahashi

**Affiliations:** First Department of Surgery, Kyoto Prefectural University of Medicine, Japan.

## Abstract

**Images:**


 © Macmillan Press Ltd., 1994

### *In vitro* reactivity and *in vivo* biodistribution of the monoclonal antibody A7 using human gastric carcinoma cell lines

N. Yamaoka, T. Yamaguchi, E. Otsuji, M. Kato, T. Kotani, K. Kitamura & T. Takahashi

*First Department of Surgery, Kyoto Prefectural University of Medicine, Kyoto, Japan.*

**Summary** The monoclonal antibody (MAb) A7 has been used to treat patients with colorectal or pancreatic carcinoma with encouraging results. We therefore determined if MAb A7 would also react with gastric carcinoma cell lines. MAb A7 reacted with seven of eight gastric carcinoma cell lines tested. The intensity of the reaction, measured by flow cytometry, was equal to that of WiDr (colon) and HPC-YS (pancreas) cell lines. In nude mice bearing xenografts of the MAb A7-reactive gastric cancer line MKN45, the percentage injected dose of MAb A7 per g of tumour tissue on day 7 was 9.79; this value was 77% of that on day 1. The *in vivo* tumour-to-blood ratio of MAb A7 was 2.77 on day 7. Therefore, MAb A7 has long-term retention at binding sites as well as a high probability, high intensity and high specificity of reactivity against gastric cancer, which make it an ideal drug carrier for immunotargeted chemotherapy and immunodiagnosis.

Many gastric cancers are now detected at an early stage, mainly because of mass screening and recent improvements in gastrofibrescopic endoscopy. Early gastric cancers now account for almost 50% of clinically detected gastric cancers in Japan (Bollschweiler *et al.*, 1993) and 95% of patients with early gastric cancer can be cured by surgery (Inoue *et al.*, 1991; Moriguchi *et al.*, 1991). Gastric cancer remains the most common carcinoma in Japan (Tominaga, 1987). Thus, there are still many advanced cases of gastric cancer that lead to death despite surgical treatment and chemotherapy. Ultrasound tomography, computerised tomography (CT) and angiography are routinely used in the staging of gastric cancer. But their sensitivity and specificity limit their ability in preoperative staging and evaluating the completeness of a resection.

One possible approach to overcome the limitations of both current chemotherapy and present diagnostic techniques is to attach anti-cancer agents or radionuclei to monoclonal antibodies (MAbs) against various solid tumours, so that a drug or radioactive isotope can be selectively confined to tumour cells (Tjandra *et al.*, 1989; Boeckmann *et al.*, 1990; Elias *et al.*, 1990; Jan-Erik *et al.*, 1990; Hani *et al.*, 1991). We have developed MAb A7 against a human colonic carcinoma (Kotanagi *et al.*, 1986) and have covalently combined MAb A7 with the anticancer antibiotic neocarzinostatin (NCS) to produce a MAb-drug conjugate, A7-NCS. A7-NCS has a significant anti-tumour effect on grafted human colonic carcinoma in nude mice. We have already treated patients with colorectal carcinoma with A7-NCS and have obtained encouraging results. An early A7-NCS trial has shown that three of eight patients with post-operative liver metastasis showed evidence of tumour reduction on CT scan, and three obtained pain relief without severe adverse effects (Takahashi *et al.*, 1988). Furthermore, A7-NCS treatment prolonged survival time when compared with conventional chemotherapy in the patients with liver metastasis (median survival times: A7-NCS group, 328 days; conventional chemotherapy group, 128 days; $P<0.05$) (Takahashi *et al.*, 1993). Furthermore, in our recent study, MAb A7 was shown to react with 77% of human pancreatic carcinoma cell lines as well as with human colonic carcinoma (Otsuji *et al.*, 1990, 1993). This suggests that MAb A7 may react with several kinds of carcinoma, e.g. gastric carcinoma, and that MAb A7-antitumour agent conjugates may have a favourable effect on those carcinomas.

Correspondence: N. Yamaoka, First Department of Surgery, Kyoto Prefectural University of Medicine, Kawaramachi Hirokoji, Kamigyo-ku, Kyoto 602, Japan.
Received 15 December 1993; and in revised form 19 April 1994.

In this study, we investigated the *in vitro* reactivity of MAb A7 with eight gastric carcinoma cell lines using flow cytometry, and also examined the *in vivo* biodistribution of MAb A7 using human gastric cancer xenografts in nude mice in order to determine their therapeutic and diagnostic usefulness in a preclinical model.

### Materials and methods

### *Cell lines*

The human gastric carcinoma cell lines MKN1 (adenosquamous carcinoma), MKN28 (moderately differentiated adenocarcinoma), MKN45 (poorly differentiated adenocarcinoma), MKN74 (well-differentiated adenocarcinoma), NUGC2 (moderately differentiated adenocarcinoma), NUGC3 (moderately differentiated adenocarcinoma), AZ521 (moderately differentiated adenocarcinoma) and KATO III (signet ring cell carcinoma), the human colonic carcinoma cell line WiDr and the human pancreatic carcinoma cell line HPC-YS were used in this study. All of the gastric carcinoma cell lines and WiDr were generously provided by JCRB (Japanese Cancer Research Resources Bank, Tokyo). HPC-YS was a kind gift from N. Yamaguchi (Department of Research, Institute of Neurology and Geriatrics, Kyoto Prefectural University of Medicine) (Yamaguchi *et al.*, 1990). All the cell lines were maintained in RPMI-1640 medium supplemented with 10% heat-inactivated fetal bovine serum (FBS) (Flow Laboratories, Rockville, MD, USA).

### *Monoclonal antibodies*

The murine monoclonal antibody MAb A7 was developed against human colonic carcinoma as described previously (Kotanagi *et al.*, 1986). MAb A7 has been shown to react with a 45 kDa surface glycoprotein of human colonic carcinomas (Kitamura *et al.*, 1989) and 77% of the human pancreatic carcinoma cell lines tested, including HPC-YS. The A7 antigen loses antigenic activity after sodium periodate, pronase and ficin treatments. MAb A7 does not react with normal gastric mucosa, erythrocytes, peripheral lymphocytes or ileal mucosa, and has weak reactivity with 10% of colon mucosa specimens (Kotanagi *et al.*, 1986). Normal mouse IgG was purchased from Boehringer Mannheim Biochemicals (Mannheim, Germany).

### *Cell fixation and staining*

For immunostaining, $1 \times 10^6$ cells were incubated in 2 ml of phosphate-buffered saline (PBS) containing $30\,\mu g\,ml^{-1}$ MAb

A7 and 1% bovine serum albumin (BSA) (Sigma, St Louis, MO, USA) for 1 h at 37°C, washed twice with PBS and fixed in 50% methanol at −20°C for 15 min. After fixation, cells were washed and incubated for 30 min with fluorescein isothiocyanate (FITC)-labelled rabbit anti-mouse antibody (Dako, Denmark) diluted 1:40 in PBS containing 1% BSA. The cells were washed once in PBS, resuspended in 10 $\mu g\ ml^{-1}$ propidium iodide (PI) (Sigma) and 1 $mg\ ml^{-1}$ RNAse A (Sigma) in PBS, and incubated at room temperature for 30 min prior to flow cytometry.

### *Flow cytometry*

Samples were analysed on a FACScan flow cytometer (Becton Dickinson, USA) equipped with a 15 mW air-cooled 488 nm argon ion laser. Green fluorescence was collected after a 530 nm bandpass filter. PI emissions were filtered through a 585 nm bandpass filter. Electronic compensation was used to remove residual spectral overlap. The reactivity to MAb A7 was measured by FITC fluorescence, and DNA content was measured simultaneously by PI fluorescence. All data were stored in list mode and analysed with a CellFit operating system (Becton Dickinson) and Lysis operating system (Becton Dickinson). Doublets and debris were gated out from the red peak *vs* integral dot plot. Data from 30,000 events were collected in the final gated histogram.

### *Preparation of radiolabelled MAbs*

MAb A7 and normal mouse IgG labelled with $^{125}I$ (IMS 30, Amersham Japan) were obtained by the chloramine-T method (Hunter & Greenwood, 1962). The iodinated MAb A7 was separated from excess reactants by gel filtration on a Sephadex G25 column (Pharmacia, Sweden). The specific activities of the [$^{125}I$]MAb A7 and $^{125}I$-labelled normal mouse IgG were 1.2 to 1.3 $\mu Ci\ \mu g^{-1}$ respectively.

### *Tumour xenograft*

Cultured MKN45 cells were harvested by ethylenediaminetetraacetic acid (EDTA) treatment, washed in PBS and resuspended in PBS. Approximately $1 \times 10^7$ viable cells were injected subcutaneously into the left flank of 8-week-old athymic nude mice (Balb/c, *nu*/*nu*, male, mean body weight approximately 21 g) (SLC, Shizuoka, Japan). A tumour mass was detected in each mouse injected with MKN45 cells (tumour weight 226 ± 32 mg, diameter approximately 8 mm). MKN1 cells were also inoculated into the mice by the same method as a negative control (tumour weight 187 ± 28 mg, diameter approximately 7 mm).

### In vivo *distribution of radiolabelled antibodies*

The human gastric cancer-bearing nude mice were injected intravenously with 0.7 $\mu Ci$ of either $^{125}I$-labelled A7 or $^{125}I$-labelled normal mouse IgG. Four mice from each group were sacrificed and dissected on days 1, 3, 5 and 7. After dissection, the tumours, blood, lungs, heart, liver, spleen, pancreas, stomach, small intestine, colon and kidneys were weighed. Radioactivity in the tissues was measured using a gamma scintillation counter (Auto-Gamma 5000, Packard, IL, USA). The results from the various tissues were expressed as c.p.m. $g^{-1}$ and compared with those for normal mouse IgG. To compare the specific localisation of MAb A7 in the tumour with that in the normal tissues, the ratio of radioactivity in these tissues to that in the blood was calculated. These ratios were derived by dividing the radioactivity in the various tissues on a per weight basis by that in the total blood on a per weight basis. The Student's *t*-test was used to check for statistically significant differences. A *P*-value of less than 0.05 was considered to be statistically significant.

The distribution of the antibodies in the tumours was also measured by the same method using nude mice harbouring MKN1 tumours.

### Results

### In vitro *reactivity of gastric cancer cell lines*

MAb A7 reacted with seven of the eight gastric carcinoma cell lines tested (87%) (Figure 1). Only MKN1, an adenosquamous carcinoma, did not react with MAb A7. The mean fluorescence cell intensity of each of the seven gastric cancer cell lines was equal to that of the colonic cancer cell line WiDr and the pancreatic cancer cell line HPC-YS.

As shown in Figure 2, the cell surface of MKN45 and KATO III was stained with MAb A7 FITC immunofluorescence staining and a cell nucleus was dyed with PI. By contrast, in the case of MKN1, the cell surface was not stained, only a cell nucleus was dyed by PI.

### In vivo *distribution of radiolabelled antibodies*

More radiolabelled MAb A7 accumulated in the grafted MKN45 tumours than in the blood after day 3, and high radioactivity was maintained until day 7 (Figure 3). On the other hand, less radiolabelled normal mouse IgG accumulated in the tumour than in the blood at all times. In contrast, in mice harbouring MKN1 both radiolabelled IgG and MAb A7 localised less in the tumour than in the blood at all points of study (Figure 4).

**Figure 1** *In vitro* reactivity of gastric cancer cell lines with the anti-human colon carcinoma antibody MAb A7. MAb A7 reacted with seven of eight gastric carcinoma cell lines (87%). Only MKN1 was not recognised by MAb A7. The mean intensity of cell fluorescence of the gastric cancer cell lines was equal to that of WiDr and HPC-YS.

**Figure 2** Immunofluorescence staining of the gastric cancer cell lines MKN45, KATO III and MKN1 by MAb A7. $1 \times 10^6$ cells were reacted with 30 $\mu g\ kg^{-1}$ MAb A7 as described in the Materials and methods section. The cell surface of MKN45 and KATO III stains with MAb A7 and a cell nucleus was dyed with PI. By contrast, in the case of MKN1, the cell surface was not stained, only a cell nucleus was dyed by PI. Left: MKN45. Middle: KATO III. Right: MKN1.

**Figure 3** The distribution of $^{125}$I-labelled MAb A7 in the blood (open symbols) and tumours (closed symbols) of mice bearing MKN45 tumours after intravenous injection. Human gastric cancer MKN45-bearing nude mice were injected intravenously with 0.7 μCi of either $^{125}$I-labelled A7 (circles) or $^{125}$I-labelled normal mouse IgG (triangles). Four mice from each group were sacrificed and dissected on days 1, 3, 5 and 7. The radioactivity in the tumour and in the blood was measured using a gamma scintillation counter and expressed as c.p.m. g$^{-1}$ tissue.

**Figure 4** Distribution of $^{125}$I-labelled MAb A7 in the blood and tumours of mice bearing MKN1 tumours after the intravenous injection. The amount of radioactivity in the blood and in the tumours of mice harbouring MKN1 is shown. Mice were injected and radioactivity was measured as in Figure 3. Symbols as in Figure 3.

The MAb A7 tumour-to-blood ratio rose progressively to 2.77 ± 0.19 on day 7 (Figure 5). In contrast, the values in normal organs were all below 0.38 ± 0.09 and did not change over time. The normal mouse IgG tumour-to-blood ratio ranged from 0.25 ± 0.07 to 0.32 ± 0.06 during this experiment. The difference in tumour–blood ratio between the two groups was statistically significant.

### Discussion

We have used the monoclonal antibody (MAb) A7 as a drug or radioactive isotope carrier to treat colorectal and pan-

**Figure 5** Tissue–blood ratios of $^{125}$I-labelled MAb A7 in nude mice bearing human gastric cancer xenografts. Tissue–blood ratios of $^{125}$I-labelled MAb A7 and $^{125}$I-labelled normal mouse IgG on days 1, 3, 5 and 7 are shown. These ratios were derived from dividing the radioactivity in the various tissues on a per weight basis by that in the total blood on a per weight basis. **a**, MKN45 tumour-bearing mice. **b**, MKN1 tumour-bearing mouse. Mice were injected and radioactivity was measured as described in the Materials and methods section.

creatic cancers. In this study, we evaluated the possibility of using MAb A7 in gastric cancer treatment and diagnosis. If Mab A7 is to be useful for immunotargeted chemotherapy and for imaging with radiolabelled monoclonal antibodies in patients with gastric carcinoma, it will be necessary to document that MAb A7 has a high probability, high intensity and high specificity of binding to gastric cancer cells, as well as long-term retention at the binding sites.

Histological heterogeneity is a characteristic feature of gastric cancer that differs from colorectal cancer. In this report, we studied the reactivity of MAb A7 with various histological types of cancer cell lines. MAb A7 reacted with the common types of gastric cancer cells regardless of the degree of differentiation, but it did not react with the adenosquamous carcinoma cell line, MKN1. It has been previously reported that MAb A7 does not react with a squamous cell carcinoma cell line, KB-2 (Otsuji *et al.*, 1992). However, the common types are the absolute majority of all gastric carcinomas. Therefore, MAb A7 has a high probability of reacting with gastric carcinoma.

According to our flow cytometry measurements, the *in vitro* intensity of the reactivity with the common types of gastric carcinoma cell lines was as high as that with the colonic carcinoma cell line WiDr and the pancreatic carcinoma cell line HPC-YS. Moreover, the *in vivo* percentage injected dose per g of tumour tissue of MAb A7 to MKN45 on day 7 was 9.79. Those of the colonic carcinoma cell line Colon 6 and the pancreatic carcinoma cell line HPC-YS on day 8 were 8.18 and 3.3 respectively (Takahashi, 1985; Otsuji *et al.*, 1992). These findings indicate that MAb A7 has a high intensity of reactivity with gastric carcinoma, similar to its reactivity with colonic and pancreatic carcinoma.

The *in vivo* tumour-to-blood ratio of MAb A7 is a good index of the specificity of MAb A7. The tumour-to-blood ratio of MKN45 on day 7 was 2.77, 8.65 times that of normal mouse IgG. The ratio in the colonic carcinoma cell line Colon 6 was 2.38 on day 8 and was 2.04 in the pancreatic carcinoma cell line HPC-YS (Takahashi, 1985; Otsuji et al., 1992). The tissue-to-blood ratio of each of the normal organs was significantly lower than the tumour-to-blood ratio, and no difference between MAb A7 and normal mouse IgG was found. These findings indicate that MAb A7 has a high specificity for gastric carcinoma, similar to that for colonic and pancreatic carcinomas. Reintgen *et al.* (1983) have reported that an antibody tumour–blood ratio must be greater than 2–10 for reliable tumour imaging, and Tsuda *et al.* (1990) have successfully performed immunoscintigraphy of tumour xenografts in nude mice after injection of an $^{131}$I-labelled MAb, which achieves a tumour tissue–blood ratio of 3.1. Moreover, the low distribution of A7 to normal organs may lead to a low incidence of side-effects during therapy and low background during diagnostic scintigraphy.

The *in vivo* percentage injected dose of MAb A7 per g of tumour tissue on day 7 was 77% of that on day 1. This shows that MAb A7 is retained at the binding sites, and means that the dose of the anti-tumour agent which is carried out by MAb A7 could be decreased, and would not have to be administered frequently, which may also reduce the incidence of side-effects.

Recently, Sickle-Santanello *et al.* (1987) have developed a hand-held gamma detection probe for intraoperative use. The aim of radical surgery for gastric cancer is to provide an uninvolved surgical margin and lymph nodes. However, in practice, tissues dissected during this procedure may not be involved pathologically. A radioimmuno-guided procedure using MAbs may be useful for determining the extent of a gastric cancer.

In conclusion, MAb A7 has the characteristics of an ideal drug carrier for immunotargeted chemotherapy and radiolabelled monoclonal antibody imaging of gastric cancer. We expect that MAb A7 will prove clinically useful for the treatment of gastric carcinoma.

This work was supported in part by a Grant-in-Aid for Cancer Research from the Ministry of Education, Science and Culture, Japan, and a grant from the Ministry of Health and Welfare, Japan.

### References

BOECKMANN, W., BAUM, R.P., SCHULDES, H., KRAMER, W., HERTEL, A., BAEW-CHRISTOW, T., HANKE, P., JONAS, D. & HOR, G. (1990). Tumour imaging of bladder carcinomas and their metastasis with $^{111}$indium-labelled monoclonal anti-CEA antibody BW 431/26. *Br. J. Cancer*, **62**, 81–84.

BOLLSCHWEILER, E., BOETTCHER, K., HOELSCHER, A.H., SASAKO, M., KINOSHITA, T., MARUYAMA, K. & SIEWERT, J.R. (1993). Is the prognosis for Japanese and German patients with gastric cancer really different? *Cancer*, **71**, 2918–2925.

ELIAS, S.J., KLINEL, K.W., DILLMANN, R.O., ROBB, J.A., WALKER, L.E. & TIMMS, R.M. (1990). Monoclonal antibody KS1 4-methotrexate conjugate in patients with non-small cell lung carcinoma. *Antibody Immunoconjugates Radiopharm.*, **3**, 60.

HANI, H.A., ALAN, N.S., DEBRA, G.W., CELESTIA, S.H., JUDITH, A.O. & MICHAEL, W.U. (1991). Scintigraphic detection of gastric and pancreatic carcinomas with In-111 ZCE 025 monoclonal antibody. *World J. Surg.*, **15**, 122–127.

HUNTER, W.M. & GREENWOOD, F.C. (1962). Preparation of iodine, $^{131}$I-labelled human growth hormone of high specific activity. *Nature*, **194**, 495–496.

INOUE, K., TOBE, T., KAN, N., NIO, Y., SAKAI, M. & TAKEUCHI, E. (1991). Problems in the definition and treatment of early gastric cancer. *Br. J. Surg.*, **78**, 818–821.

JAN-ERIK, F., ANN-KARI, L. & HAKAN, M. (1990). Pharmacokinetics of the mouse monoclonal antibody 17-1A in cancer patients receiving various treatment schedules. *Cancer Res.*, **50**, 4866–4871.

KITAMURA, K., TAKAHASHI, T., YAMAGUCHI, T., YOKOTA, T., NOGUCHI, A., AMAGAI, T. & IMANISHI, J. (1989). Immunochemical characterization of the antigen recognized by the murine monoclonal antibody A7 against human colorectal cancer. *Tohoku J. Exp. Med.*, **157**, 83–93.

KOTANAGI, H., TAKAHASHI, T., MASUKO, T., HASHIMOTO, Y. & KOYAMA, K. (1986). A monoclonal antibody against human colon cancers. *Tohoku J. Exp. Med.*, **148**, 353–360.

MORIGUCHI, S., OKADA, T., HAYASHI, Y., NOSE, Y., MAEHARA, Y., KORENAGA, D. & SUGIMACHI, K. (1991). Death due to recurrence following curative resection of early gastric cancer depends on age of the patient. *Br. J. Cancer*, **64**, 555.

OTSUJI, E., TAKAHASHI, T., YAMAGUCHI, T., YAMAGUCHI, N. & IMANISHI, J. (1990). Specific cytotoxic effect of neocarzionstatin conjugated to monoclonal antibody A7 on human pancreatic carcinoma. *Gastroenterol. Jpn.*, **25**, 244–248.

OTSUJI, E., YAMAGUCHI, T., YAMAOKA, N., YAMAGUCHI, N., IMANISHI, J. & TAKAHASHI, T. (1992). Biodistribution of monoclonal antibody A7 and its F(ab')$_2$ fragment in athymic nude mice bearing human pancreatic carcinoma. *J. Surg. Oncol.*, **50**, 173–178.

OTSUJI, E., YAMAGUCHI, T., YAMAOKA, N., KITAMURA, K., YAMAGUCHI, N., IMANISHI, J. & TAKAHASHI, T. (1993). Increased antitumor effect of neocarzinostatin conjugated to monoclonal antibody A7 on human pancreatic carcinoma grafted in nude mice. *Antibody Immunoconjugates Radiopharm.*, **6**, 177–183.

REINTGEN, D.S., SIMIZU, K., COLEMAN, E., BRINER, W., KITZMLILLER, J., EISENBARTH, G. & SEIGLER, H.F. (1983). Immunodiagnosis of tumors *in vivo* using radiolabeled antibody A285. *J. Surg. Oncol.*, **23**, 205–211.

SICKLE-SANTANELLO, B.J., O'DWYER, P.J., MOJZISIK, C., TUTTLE, S.E., HINKLE, G.H., ROUSSEAU, M., SCHROM, J., COLCHER, D., THURSTON, M.O., NIERODA, C., SARDI, A., FARRAR, W.B., MINTON, J.P. & MARTIN, Jr, E.W. (1987). Radioimmunoguided surgery using the monoclonal antibody B72.3 in colorectal tumors. *Dis. Colon Rectum*, **30**, 761–764.

TAKAHASHI, M. (1985). *In vivo* localization of monoclonal antibody A7 in human colon cancer inoculated into Nude Mice. *Akita J. Med.*, **12**, 481–494.

TAKAHASHI, T., YAMAGUCHI, T., KITAMURA, K., SUZUYAMA, H., HONDA, M. & HASHIMOTO, Y. (1988). Clinical application of monoclonal antibody–drug conjugates for immunotargeting chemotherapy of colorectal carcinoma. *Cancer*, **61**, 881–888.

TAKAHASHI, T., YAMAGUCHI, T., KITAMURA, K., NOGUCHI, A., HONDA, M. & OTSUJI, E. (1993). Follow-up study of patients treated with monoclonal antibody–drug conjugate: report of 77 cases with colorectal cancer. *Jpn J. Cancer Res.*, **84**, 976–981.

TJANDRA, J.J., PIETERSZ, G.A. & CUTHBERTON, A.M. (1989). Phase I clinical trial of drug–monoclonal antibody conjugates in patients with advanced colorectal carcinoma: a preliminary report. *Surgery*, **106**, 533–545.

TOMINAGA, S. (1987). Decreasing trend of stomach cancer in Japan. *Jpn J. Cancer Res.*, **78**, 1–10.

TSUDA, T., KOSHIBA, H., USUI, T., KUBOTA, M., KIKUCHI, K. & MORITA, K. (1990). Radioimmunoscintigraphy of human pancreatic carcinoma xenografts in nude mice with $^{131}$I-labeled monoclonal antibody. *Ann. Nucl. Med.*, **4**, 71–74.

YAMAGUCHI, N., YAMAMURA, Y., KOYAMA, K., OTSUJI, E., IMANISHI, J. & ASHIHARA, T. (1990). Characterization of new human pancreatic cancer cell lines which propagate in a protein-free chemically defined medium. *Cancer Res.*, **50**, 7008–7012.